# Biased Ligands Differentially Shape the Conformation of the Extracellular Loop Region in 5-HT_2B_ Receptors

**DOI:** 10.3390/ijms21249728

**Published:** 2020-12-20

**Authors:** Katrin Denzinger, Trung Ngoc Nguyen, Theresa Noonan, Gerhard Wolber, Marcel Bermudez

**Affiliations:** Institute of Pharmacy, Freie Universität Berlin, Königin-Luise-Strasse 2-4, 14195 Berlin, Germany; k.denzinger@fu-berlin.de (K.D.); trungngoc.nguyen@fu-berlin.de (T.N.N.); theresa.noonan@fu-berlin.de (T.N.); gerhard.wolber@fu-berlin.de (G.W.)

**Keywords:** GPCR, biased signaling, molecular dynamics, serotonin receptors, virtual screening, conformational descriptor, pharmacophore, drug design

## Abstract

G protein-coupled receptors are linked to various intracellular transducers, each pathway associated with different physiological effects. Biased ligands, capable of activating one pathway over another, are gaining attention for their therapeutic potential, as they could selectively activate beneficial pathways whilst avoiding those responsible for adverse effects. We performed molecular dynamics simulations with known β-arrestin-biased ligands like lysergic acid diethylamide and ergotamine in complex with the 5-HT_2B_ receptor and discovered that the extent of ligand bias is directly connected with the degree of closure of the extracellular loop region. Given a loose allosteric coupling of extracellular and intracellular receptor regions, we delineate a concept for biased signaling at serotonin receptors, by which conformational interference with binding pocket closure restricts the signaling repertoire of the receptor. Molecular docking studies of biased ligands gathered from the BiasDB demonstrate that larger ligands only show plausible docking poses in the ergotamine-bound structure, highlighting the conformational constraints associated with bias. This emphasizes the importance of selecting the appropriate receptor conformation on which to base virtual screening workflows in structure-based drug design of biased ligands. As this mechanism of ligand bias has also been observed for muscarinic receptors, our studies provide a general mechanism of signaling bias transferable between aminergic receptors.

## 1. Introduction

G protein-coupled receptors (GPCRs) are involved in the regulation of a plethora of physiological processes, and about 35% of currently marketed therapeutics directly target GPCRs [1]. In the last decade, structural and biophysical data helped to revisit our understanding of GPCR activation and revealed a surprisingly complex pharmacology [2] including several dimensions of spatiotemporal signaling [3,4,5,6,7,8]. One important aspect of this complexity is the preference of a certain ligand–receptor complex for one intracellular transducer protein over another, commonly referred to as biased signaling or functional selectivity [9]. Signaling bias can contribute to a desired effect or trigger adverse reactions [10,11,12]. Its rational design remains challenging, but without any doubt, biased ligands bear a vast therapeutic potential to be uncovered [13,14,15]. Mechanistic understanding of ligand-triggered receptor activation is key to develop drugs with biased signaling properties.

**Figure 1:**
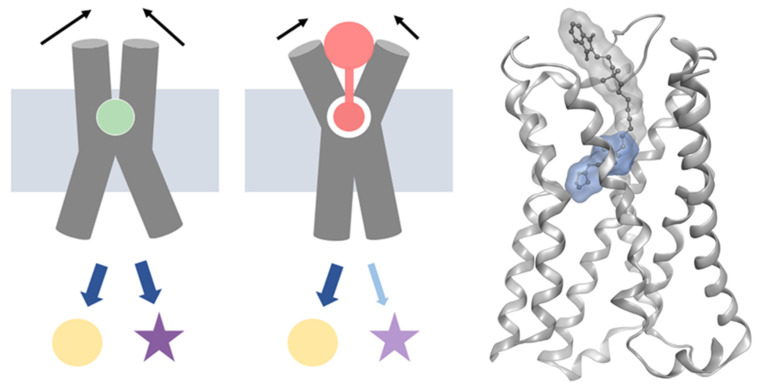
A schematic representation of the general activation mechanism illustrates the full signaling repertoire of a classical orthosteric agonist (left, green circle) and the restricted conformational freedom by extended molecular motives (middle, salmon shape) resulting in biased signaling. One example of such extended (bitopic) ligands is iper-8-phth, which is shown on the right in its proposed binding mode at the M_1_ receptor [22]. While the iperoxo building block (blue surface) binds to the orthosteric binding pocket, the extended molecular structure (grey shape) spatially interferes with extracellular loop regions.

Current activation models are based on allosteric coupling of the ligand binding site with intracellular receptor regions allowing for binding of different intracellular binding partners like G proteins or β-arrestin. Activation upon ligand binding causes long-range conformational changes within the receptor, resulting in an outward movement of the intracellular part of transmembrane domain 6 (TM6) to enable binding of intracellular transducers and subsequent release of second messenger molecules [16]. Reciprocally, G-protein binding causes a closure of the binding pocket by drawing together extracellular loops 2 and 3 (ECL2 and ECL3, respectively), trapping the ligand within the binding pocket and stabilizing the ternary GPCR complex [17]. Due to the dynamic nature of GPCRs, multiple receptor conformations exist between the fully active and inactive state, and each ligand stabilizes a distinct ensemble of receptor conformations [18,19]. Thus, ligands that extend into the extracellular vestibule can affect the degree of binding pocket closure via their interaction patterns with extracellular epitopes. This in turn might influence the degree of intracellular opening resulting in a differential recruitment of signal transducers (Figure 1) [20]. The relationship between ligand interaction patterns at the extracellular loop region, ECL closure, and resulting pharmacological functions has recently been studied with bitopic ligands at muscarinic receptors (SI Figure S1) [21,22]. These findings show that ligand-dependent conformational constraints at the extracellular binding site can be linked to a restriction of the muscarinic receptor’s signaling repertoire.

The present study follows the hypothesis that this mechanism for biased signaling is transferable to other aminergic receptors. Given the availability of structural and functional data, we focus on the 5-HT_2B_R and its co-crystallized, nature-derived, β–arrestin-biased ligands lysergic acid diethylamide (LSD) and ergotamine [23,24]. We compare their influence on extracellular loop dynamics with the unbiased endogenous ligand serotonin (5-HT) by means of molecular dynamics (MD) simulations and dynamic pharmacophores. The analysis of additional biased 5-HT_2B_R ligands from the *BiasDB*, a publicly available database for biased GPCR ligands [25], and recent structural data of the 5-HT_1A_R, 5-HT_1B_R, and dopamine D_2_ receptor support our findings [23,26,27,28]. Given the existence of pathway-specific binding site conformations, we discuss implications for structure-based approaches in drug design, providing a route to virtually screen for ligands with a specific signaling profile by using the corresponding receptor conformation as starting point.

## 2. Results

### 2.1. Extended Binding Modes of LSD and Ergotamine

**Figure 2:**
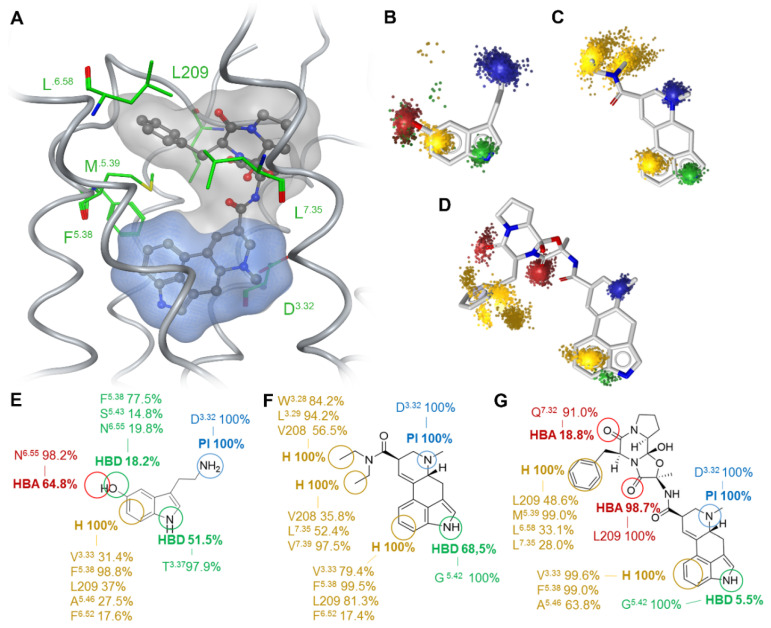
Extended binding modes of biased 5-HT_2B_R agonists. Close-up view of the ligand binding site of 5-HT_2B_R in complex with ergotamine indicating the most important residues in green (**A**). The blue surface represents the common core structure of all studied ligands, while the grey surface indicates the extended molecular structure. Dynamic pharmacophores of 5-HT (**B**,**E**), LSD (**C**,**F**), and ergotamine (**D**,**G**) indicate common interactions for 5-HT_2B_R agonists and additional interactions likely accounting for ligand-specific effects. The percentages in panel E-G refer to the feature occurrence over 200 ns of MD simulation, while the three-dimensional dynamic pharmacophores (**B**–**D**) illustrate the spatial distribution of interaction features in the trajectory. The color code is composed of blue for positive ionizable areas (*PI*), yellow for hydrophobic contacts (*H*), green for hydrogen bond donors (*HBD*), and red for hydrogen bond acceptors (*HBA*).

We analyzed and compared the binding modes of the unbiased endogenous agonist 5-HT, the β-arrestin-biased agonist LSD, and the agonist with the strongest β-arrestin bias reported to date, ergotamine [23]. The common structural part of the three investigated ligands consists of an aromatic indole ring with a 3-ethylamine group. In LSD and ergotamine, this moiety is embedded in the lysergic acid ring system, which is a precursor for ergoline alkaloids. Lysergic acid is a chiral compound with two stereocenters at C5 and C8. While the C5 atom exists in a stable R-configuration, the C8 stereocenter can epimerize under acidic or basic conditions due to the double bond between C9 and C10. The isomer with C8-(S) configuration is known as iso-lysergic acid [29]. LSD and ergotamine both manifest the C8-(R) configuration. Ergotamine is larger than LSD and 5-HT and the lysergic acid forms an amide bond with a tripeptide moiety whereas in LSD the amide bond consists of lysergic acid and diethylamine. The binding orientations of LSD and ergotamine in the 5HT_2B_R structures (PDB entries: 5TVN and 4IB4 [23,24]) are quite similar, in which the indole moiety is oriented towards transmembrane TM4 and TM5 (Figure 2A). For 5-HT, we selected a docking pose that resembles the orientation of the common substructure. The tripeptide moiety of ergotamine and the diethylamide of LSD are located in the extended binding pocket [23,24] that is part of the extracellular receptor region and point towards ECL2.

We performed all-atom MD simulations of the three ligand–receptor complexes for 200 ns in triplicates to obtain insights into ligand–receptor dynamics. Dynamic pharmacophore analysis resulted in superfeatures, which represent the dynamic ligand–receptor interaction pattern characterized by feature occurrence and spatiotemporal feature distribution [30]. Binding at the inner core region of 5-HT_2B_R, we identified four superfeatures for 5-HT (Figure 2B,E). The first is a positive charge, situated around the primary amine and present for every frame of the MD trajectory. The primary amine is protonated under physiological condition and builds a salt bridge to D^3.32^ which is oppositely charged. As this interaction is mandatory for activation of aminergic receptors [31], its constant appearance validates the binding pose of 5-HT. The second feature is a lipophilic contact centered on the phenyl ring of the indole moiety. Five different residues are involved in this contact, amongst them F^5.38^ for almost the whole trajectory, L209 in ECL2, V^3.33^ and A^5.46^ in around 30% of the simulation time. F^6.52^ shows the least frequent occurrence at around 17%. The nitrogen of the electron-rich aromatic indole is embedded in a hydrogen bond donor superfeature, forming a hydrogen bond to T^3.37^ during 50% of the trajectory. The fourth superfeature reveals the hydroxyl group at position 5 to act as a simultaneous hydrogen bond donor and acceptor with N^6.55^, F^5.38^ and S^5.43^. All four features are spherical and evenly distributed indicating a plausible binding orientation of 5-HT. The interactions of LSD are represented by five superfeatures (Figure 2C,F): A positive charge is spherically placed around the tertiary amine of the ergoline ring system, which again builds a salt bridge to D^3.32^. The nitrogen of the aromatic indole ring is surrounded by a hydrogen bond donor feature which builds a hydrogen bond to G^5.42^ during 68% of the trajectory. The six-membered ring of the indole moiety is located within a stable lipophilic contact with L209, V^3.33^, F^5.38^, and F^6.52^. The diethylamide moiety binds in the extended binding pocket of 5HT_2B_R [24] which leads to the extracellular receptor region. We observed two stable lipophilic contacts representing the diethylamide group. One is oriented towards TM3 and interacts with W^3.28^, L^3.29^, and V208 in ECL2. The second one is oriented towards TM7, interacting with L^7.35^, V^7.39^, and V208 in ECL2. The shape of the lipophilic contacts is rather elongated, which is due to the high flexibility of the freely rotatable bonds of the two ethyl groups. Of the three ligands under investigation, ergotamine extends the furthest into the extended binding pocket, showing a multitude of contacts to residues within ECL2 and the top of helix six and seven (Figure 2D,G). Within the inner receptor core, it displays a similar superfeature pattern when compared to 5-HT or LSD. These comprise again the positive charge area for the salt bridge between the tertiary amine and D^3.32^ in every frame, lipophilic contacts for the benzol part of the ergoline ring system to F^5.38^ and V^3.33^ in 99% of all frames and A^5.46^ in 64%. A hydrogen bond donor feature around the indole nitrogen which builds a hydrogen bond to G^5.42^ can only be observed in around five percent of the frames. For the tripeptide moiety that is situated in the extracellular receptor region, we observed two hydrogen bond acceptor features. The first is located around the oxygen of the amide connecting alanine and phenylalanine of ergotamine, which forms a stable hydrogen bond to the L209 backbone nitrogen. The second is distributed around the oxygen of the amide that connects phenylalanine and proline and Q^7.32^ in 17% of all frames. The phenyl group of phenylalanine is situated in the middle of a flexible lipophilic contact, interacting with L290, L^7.35^, M^5.39^, and L^6.58^. The interaction with M^5.39^ is preserved over the whole simulation, whereas the phenyl ring shifts from interacting with L^7.35^ and L^6.58^ to interacting with L209 after around 100 ns of simulation. Therefore, the phenyl ring of ergotamine can adopt two distinct conformations, which is visible in the superfeature space representation (Figure 2D). All three ligands thus share a positive charge and the lipophilic contact on the six-ring of the indole moiety. The hydrogen bond donor interactions around the indole nitrogen are present in all three simulations, but the interaction partner or frequency is different. 5-HT interacts with T^3.37^ in helix three in 50%, LSD with G^5.42^ in helix five in 68.6% and ergotamine with G^5.42^ in 5.5% of the simulation. Due to the size of the molecules, LSD and especially ergotamine interact more frequently with extracellular loop regions for which LSD shows only hydrophobic contacts, whereas ergotamine shows hydrogen bonds in addition to the hydrophobic contacts. LSD has two stereocenters at C5 and C8 resulting in four possible isomers, of which only the C8 isomers occur in nature: (*5R,8R*)-LSD and (*5R,8S*)-LSD [29]. Only the latter isomer is psychoactive, which highlights the influence of the C8 stereocenter on the pharmacological characteristics. Since lisuride, a competitive antagonist for 5-HT_2B_R [32], has been co-crystallized with the receptor (PDB entry: 6DRX) [27], lisuride is best suited for direct comparison with LSD. Lisuride differs from LSD in the (*S*) configuration at the C8 atom and a (*S*)-diethylurea substituent instead of a (*R*)-diethylamide moiety. Due to the difference at the C8 atom, the (*S*)-diethylurea substituent is oriented towards TM3 instead of TM7 (see SI Figure S2), which has been recently shown to be essential for receptor activation [27]. Since the (*R*)-diethylamide moiety of LSD shows interactions with TM7, TM3, and ECL2 (see Figure 2), we found that lisuride cannot fulfill the interaction with TM7. Since a different configuration at C8 was reported to be the major driver for the antagonistic properties of lisuride, we did not include lisuride into the dynamic analysis to ensure the comparability between our mechanistic models.

### 2.2. Ligand-Dependent Shape of the Extracellular Loop Region Is Linked to Biased Properties

Both ergotamine and LSD are biased ligands towards β-arrestin versus Gq/s of which ergotamine is the more strongly biased ligand [23]. To investigate the mechanism underlying this bias, we measured the Cα distances between L209 (ECL2) and L^6.58^ and L^7.35^, respectively, which are well-established molecular descriptors for the examination of extracellular binding pocket closure in muscarinic receptors (SI Figure S1) [21,22].

**Figure 3:**
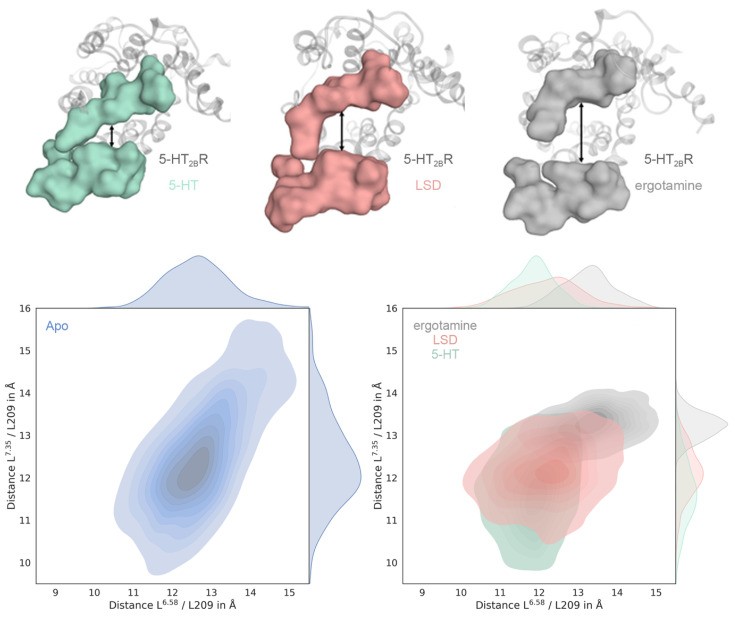
Ligand-dependent shape of the binding pocket correlates with ligand bias. The upper row shows representative frames from 200 ns MD simulations of 5-HT_2B_R with unbiased 5-HT, LSD (ß-arrestin-bias) and ergotamine (ß-arrestin-bias stronger than for LSD). The surfaces represent ECL2 and ECL3 which are mainly involved in the closure mechanism of the binding pocket. For the 5-HT structure (green), we can observe complete closure of the binding pocket, whereas the binding pocket of ergotamine (grey) is open to the biggest extent. These findings correlate with our hypothesis that the extent of closure of the extended binding pocket is linked to the degree of ligand bias. Cα distance distribution between L209 and L^6.58^ and L209 and L^7.35^ shown for the Apo structure (blue), 5-HT (green), LSD (red) and ergotamine (grey). We observe the most fluctuation for the Apo structure, followed by 5-HT, LSD and ergotamine. The latter conformationally restricts binding pocket closure the most. The medians of 5-HT, LSD and ergotamine are clearly separated, the latter being the largest, indicating the widest opening of the binding pocket for the ergotamine bound structure.

The distribution of the measured distances over the MD simulations describes the extent of binding pocket closure (Figure 3 and SI Figures S3 and S4). The 5-HT-bound structure shows the smallest median for both descriptors (Figure 3, green), followed by LSD and ergotamine clearly showing the largest distance (Figure 3, red and grey, respectively). As previously reported for muscarinic receptors, the degree of bias correlates with the shape of the extracellular loop region. The comparatively small median displayed by 5-HT supports the mechanism of binding pocket closure after receptor activation by the endogenous ligand [17,20].

We observed the widest distribution for the used conformational descriptors for the apo receptor simulation (Figure 3, blue). This is in line with our expectations, since the absence of a ligand typically means that the binding side residues are not stabilized by ligand-mediated interactions. The distance distribution at the ergotamine-bound structure is the narrowest, indicating that ergotamine stabilizes the conformation of the binding pocket in a manner that allows for less flexibility. Thus, LSD and especially ergotamine impose ligand-mediated conformational constraints on binding pocket closure which correlates with reported signaling bias via differential intracellular pathway activation [23].

### 2.3. G_q_-Biased Ligand LY266097 Shows Stronger Closure of the Extracellular Binding Pocket

**Figure 4:**
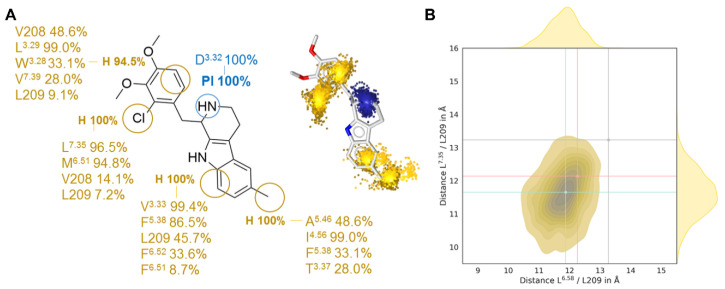
MD-based analysis of Gq-biased 5-HT_2B_R agonist LY266097. Dynophore analysis (**A**) shows two key areas for lipophilic contacts (yellow) and one positive ionizable area (blue) in the orthosteric binding pocket. The density plot (**B**) of the Cα distances between L209 and L^6.58^ and L^7.35^ indicate a stronger closure of the binding pocket compared to LSD and ergotamine-bound receptor simulations, whose medians are marked in red and grey. The median of the simulation with endogenous ligand 5-HT bound is shown in green and represents a comparably strong closure of the binding pocket to LY266097.

Using the database *BiasDB* [25], we identified additional biased ligands, amongst which cabergoline, dihydroergotamine, methylergometrine, pergolide, and 7-(4-(4-(1-Methyl-1*H*-indol-4-yl)piperazin-1-yl)butoxy)-3,4-dihydroquinolin-2(1*H*)-one (MIA) emerged as β-arrestin-biased and LY266097 as the only G_q_-biased agonist [27]. Since LY266097 shows the opposite bias to LSD and ergotamine, we hypothesized that the extracellular closing mechanism described above might not be hampered. We performed all-atom MD simulations with LY26609 (PDB entry: 6DS0 [27]) and employed the same analysis to dissect its interaction pattern to 5HT_2B_R and extracellular loop dynamics (Figure 4, SI Figure S5).

We observed the stable interaction to D^3.32^, which is mandatory for receptor activation [31]. The lipophilic portion of the tetrahydro-β-carboline core scaffold interacts in the OBP with V^3.33^, F^5.38^, F^6.52^, and L209, similar to 5-HT, LSD, and ergotamine. The methyl group creates an additional lipophilic contact to A^5.56^, I^4.56^, F^5.38^, and T^3.37^, which is not found in the other three ligands. (Figure 4A). The 2-chloro-3,4-dimethoxybenzyl substituent lies in the extracellular receptor region, interacting with diverse lipophilic residues in TM3, TM6, TM7, and ECL2, but the positioning is slightly different and allows the ECL2 and ECL3 to close the binding site in a similar manner when compared to 5-HT. As for 5-HT, LSD, and ergotamine, we measured the Cα distances between L209 and L^6.58^ and L^7.35^, respectively, as a descriptor of the influence of LY266097 on the extracellular vestibule (Figure 4B). The median of both distances lies below that of ergotamine and LSD, indicating that the pocket can close to a greater extent and that the receptor is stabilized in a different conformation. This again fits the fact that G protein binding is associated with the closing of the binding pocket. However, the differences are too marginal to be made accountable for Gq-bias of LY266097 (SI Figure S6).

### 2.4. Docking Results Strongly Depend on the Used Receptor Conformation

**Figure 5:**
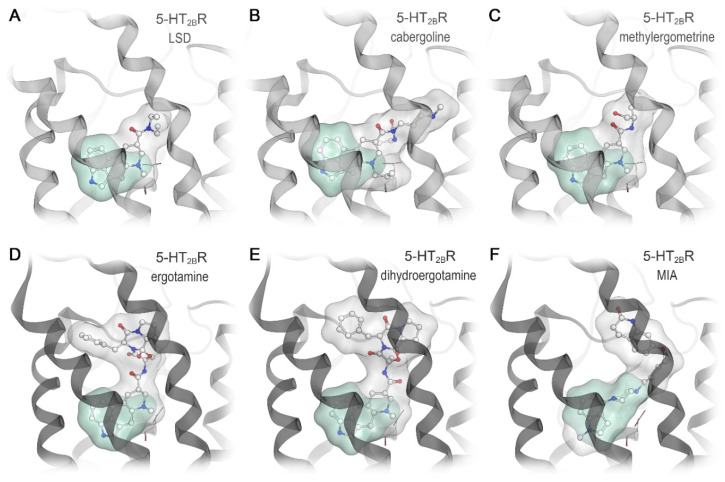
Extended binding modes are a common feature in biased 5-HT_2B_R ligands. The upper row shows LSD in its co-crystallized conformation (**A**, PDB entry: 5TVN [24]), and proposed binding modes of cabergoline (**B**) and methylergometrine (**C**) docked to the LSD-bound receptor state. The second row depicts the bitopic binding modes of ergotamine in its co-crystallized conformation (**D**, PDB entry: 4IB4 [23]), and proposed binding modes of dihydroergotamine (**E**) and MIA (**F**) docked to the ergotamine-bound receptor state. While the green surfaces indicate the serotonin substructure, the grey surfaces illustrate the extended molecule parts pointing towards the extracellular loop region.

Given the ligand-specific differences in the extracellular loop region we assessed the suitability of the LSD-bound and the ergotamine-bound receptor conformation for docking of known β-arrestin-biased agonists. We docked the biased ligands cabergoline, pergolide, methylergometrine, dihydroergotamine, and the aripiprazole derivative MIA into both crystal structures (PDB entries: 5TVN and 4IB4 [23,24]) (Figure 5). The orientation of the indole ring of LSD and ergotamine and interaction between the positively ionizable nitrogen and D^3.32^ served as a reference for evaluating the docking poses. For the smallest three ligands, cabergoline, pergolide, and methylergometrine (Figure 5A–C), we found well-fitting and similar poses in both structures with a preference for the LSD-bound conformation. For the larger ligands, dihydroergotamine and MIA, we only found plausible poses in the ergotamine-bound 5-HT_2B_R structure, which displays a higher degree of binding pocket opening (Figure 5D–F). None of the docking poses for the latter ligand group shows a plausible orientation of the 5-HT substructure and the key interaction to D^3.32^ was missing. All plausible docking poses indicate an extended binding mode with direct interaction possibilities in the extracellular loop region (Figure 5). Since cabergoline and pergolide are similar in size compared to LSD, but dihydroergotamine and MIA are more similar to ergotamine, the docking result matches the size of the ligands and the ligand-dependent receptor conformations. This highlights the importance of the receptor conformation used as a starting point for structure-based drug design.

### 2.5. Subtype-Specific Differences in Ergotamine Binding

**Figure 6:**
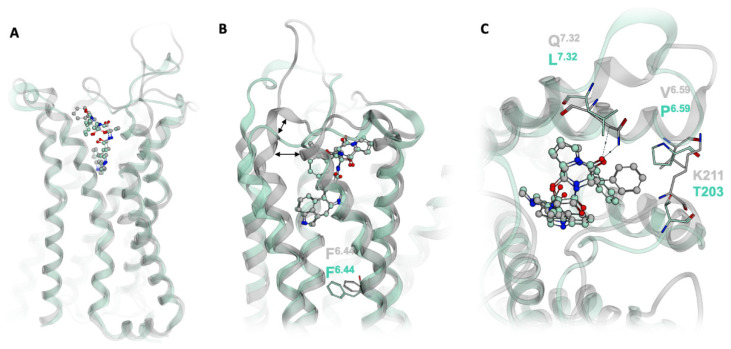
The structural basis of ergotamine’s differential bias at the 5HT_1B_R (green) and 5HT_2B_R (grey). (**A**) Structural alignment of 5HT_1B_R and 5HT_2B_R. (**B**) Due to the additional helical turn at the top of 5HT_2B_R TM5, the distance between TM5 and TM6 is shorter in 5HT_2B_R. F^6.44^ of the P-I-F motif is in the active conformation in 5HT_1B_R and in an intermediate-active conformation in 5HT_2B_R. (**C**) 5HT_2B_R forms two additional hydrophobic contacts to ergotamine, at K211 and V^6.59^. In the 5HT_2B_R TM7, ergotamine forms a hydrogen bond to Q^7.32^, which cannot be recapitulated by L^7.32^ in 5HT_1B_R.

Ergotamine binds to both the 5HT_1B_R and the 5HT_2B_R, although it shows much more pronounced β-arrestin bias at the 5HT_2B_R than the 5HT_1B_R [23]. Investigations of the crystal structure of ergotamine in complex with both receptors shows that in 5HT_1B_R, the P-I-F motif is in an active-like conformation, whereas in 5HT_2B_R, the P-I-F motif resembles an intermediate-active state (Figure 6A,B) [23]. In the 5HT_2B_R an extra helical turn at the top of TM5 causes the distance between TM5 and TM6 to shorten (Figure 6B), enabling further interactions between residues in the two helices. These additional contacts may prevent the structural rearrangements in TM6 necessary for F^6.44^ to rotate from the intermediate to the active state [23]. Furthermore, the conformational constraint on TM5 posed by the extra helical turn enables ergotamine to form additional hydrophobic contacts with K211 and V^6.59^. Interestingly, ergotamine forms a hydrogen bond to the side chain of Q^7.32^ on TM7 (Figure 2G). L^7.32^ in 5HT_1B_R (Figure 6C) is incapable of making the same interaction as ergotamine. As TM7 is involved in beta-arrestin bias [33], this may be a further structural explanation for ergotamine’s stronger bias at 5HT_2B_R compared to 5HT_1B_R. Distances in extracellular vestibule regions are emerging as powerful descriptors of ligand bias amongst different receptor types. This phenomenon is also observed in the structural difference between the ergotamine-bound forms of 5HT_1B_R and the 5HT_2B_R; the shorter distance between TM5 and TM6 in the 5HT_2B_R appear to play a role in the stronger bias of ergotamine at this receptor. Investigations into ligand selectivity for 5HT_2B_R over 5HT_1B_R found that selectivity is determined by non-conserved amino acid residues in the extracellular secondary binding pocket involving ECL2, as previously shown for other aminergic receptors [34,35].

## 3. Discussion

Given the complex pharmacology of GPCRs, it is of utmost importance for drug design to mechanistically understand how ligands transmit their chemically-encoded information through the receptor. With multiple downstream signaling pathways to be potentially activated, one focus area of GPCR research is biased signaling, i.e., how a ligand can shift the naturally imprinted signaling repertoire of a GPCR [13,15]. It has been demonstrated for muscarinic receptors (M_1_ and M_2_ receptors) that biased signaling could be achieved by interfering with the closure of the extracellular loop region upon receptor activation [21,22]. Our data contributes to that field by showing that a similar mechanism is likely the source of signaling bias at serotonin receptors. We used a combination of MD simulations and three-dimensional pharmacophore models to study receptor-ligand interactions in a dynamic manner. All studied β–arrestin-biased agonists indicated extended binding modes in which the molecule parts pointing towards the extracellular loop region showed additional and more specific interactions.

Wacker et al. described L209 in the middle of ECL2 as a key residue for LSD’s slow off-rate by forming a lid-like structure that hampers dissociation from the receptor [24]. We can also observe a stable hydrophobic contact of LSD to this residue. For ergotamine, in contrast, we observe a very stable hydrogen bond to L209 and an additional one to Q^7.32^ at the top of TM7 (Figure 2D,G). These two specific interactions for the large molecule of ergotamine could cause the wide opening of the binding pocket. Particularly the elucidation of the interaction between ergotamine and Q^7.32^ of 5HT_2B_R provides additional mechanistic understanding of the difference in ergotamine bias at the 5HT_2B_ and 5HT_1B_ (Figure 6). The dynamic pharmacophore of ergotamine indicates a high flexibility of the extracellularly located phenyl ring with at least two different binding orientations (Figure 2G). Interestingly, a similar movement of this phenyl ring can be observed in an MD simulation of ergotamine-bound 5-HT_2B_R, which is publicly available from the GPCRmd database (http://gpcrmd.org) highlighting the value of freely accessible MD trajectories [36,37]. In a similar study to the one presented here, Martí-Solano et al. performed MD simulations of the 5HT_2A_R bound to serotonin and 2-CN, a 5HT_2A_R agonist biased towards arachidonic acid production [38]. Their results correlate ligand interaction with N^6.55^ to arachidonic acid signaling, whereas interactions with S^5.46^ appear to mediate signaling through the inositol phosphate pathway. Whilst these epitopes are not the same as those we identify as responsible for 5HT_2B_R bias, investigations such as these highlight the vast array of factors contributing to biased signaling.

The conformational descriptors used for evaluating bias-related conformations of muscarinic receptors turned out to be transferable to serotonin receptors (SI Figure S1) [21]. Since the signaling profile and bias differ in muscarinic (e.g., G_i_/G_s_ and G_i_ over β-arrestin for M_2_) and serotonin receptors (mostly β-arrestin bias), we surmise that the mechanism of a conformational restriction in the ECL region leading to bias might be general, but the type of bias strongly depends on the receptor’s naturally imprinted signaling repertoire. Taking the results from MD simulations of LSD and ergotamine in comparison with 5-HT on the one hand and the missing conformational restriction in MD simulations with LY266097 on the other hand, a major role of the extracellular loop region for pathway-specific signaling is suggested. The concept of hampering binding pocket closure at extracellular receptor regions thus provides a rational way to design biased ligands by extending their molecular structure towards residues of the loop region. While this has been demonstrated for bitopic ligands of muscarinic receptors [20,21,22], our data provides evidence that similar mechanisms can harness biased signaling at serotonin receptors (5-HT_1B_R and 5-HT_2B_R). This concept is further supported by a recent crystal structure of the dopamine D_2_ receptor co-crystallized with the Parkinson’s drug bromocriptine [28]. Bromocriptine is an ergoline derivative, similar to LSD and ergotamine, and shows a strong β–arrestin-bias at D_2_ receptors. The crystal structure reports a similar binding mode and comparable interactions with residues of the extracellular loop region. Due to phylogenetic and structural cognation of aminergic GPCRs, we assume that similar mechanisms might be observed for histamine receptors or adrenoceptors as well.

Given specific receptor conformations responsible for a distinct signaling profile, it is important to choose and validate the starting structure for structure-based approaches in drug design. For example, if a virtual screening campaign aims to discover β-arrestin-biased ligands for serotonin receptors, an open conformation of the ECL region might be beneficial. Vice versa, if β–arrestin recruitment is an undesirable property, more closed receptor conformations could be advantageous. While the ergotamine-bound receptor state found plausible docking poses for all investigated ligands, docking of biased ligands was only successful for a fraction when using the LSD-bound conformation (SI Figure S7). This means that the LSD-bound receptor conformation is more restrictive, but also more specific for the pharmacological outcome. This information could also be used in pharmacophore-based virtual screening by implementing interaction features of the extracellular loop region to find more specific drugs. With more and more GPCR crystal and cryo-EM structures being solved, the right choice for a starting structure allows virtual screening workflows to be more specific regarding the pharmacological outcome of the hit molecules.

## 4. Materials and Methods

Molecular Docking: Receptors were prepared in MOE (v2019.0102; Molecular Operating Environment, Chemical Computing Group, Montreal, QC, Canada) and protonated at pH 7.4 using Protonate3D [39]. Ligands were docked into the crystal structures of the LSD-bound and ergotamine-bound 5HT_2B_R (PDB entries: 5TVN and 4IB4, respectively [23,24]) using GOLD (v.5.2; Genetic Optimization for Ligand Docking, The Cambridge Crystallographic Data Centre, UK) [40]. Binding pockets were defined as the region in which the co-crystallized ligand (LSD at the LSD-bound structure and ergotamine at the ergotamine-bound structure) plus residues within a range of 4 Å around the ligand. 25 docking poses were generated for each ligand with default settings. Poses that did not fulfil the positive ionizable interaction between the positively ionizable nitrogen and D^3.32^ were immediately discarded. Resulting poses were clustered by three-dimensional pharmacophore similarity and subsequently energy minimized in LigandScout (v4.4, Vienna, Austria) [41,42] using the MMFF94 force field [43].

Molecular Dynamics Simulations: All starting structures were prepared with MOE (v2019.0102; Molecular Operating Environment, Chemical Computing Group, Montreal, QC, Canada). The fusion proteins and antibodies were removed. Missing residues of crystal structure models of the LSD-bound 5-HT_2B_R (PDB entry: 5TVN [24], ergotamine-bound 5-HT_2B_R (PDB entry: 4IB4 [23]) and the LY266097-bound 5-HT_2B_R (PDB entry: 6DS0 [27]) were manually completed using the homology model tool and the loop model builder in MOE. The starting complexes were optimized in Maestro (v2018.3; Schroedinger, LLC, New York City, NY, USA). Simulation systems were built with periodic boundary conditions in an isothermal-isobaric ensemble and placed in an orthorhombic box using SPC water molecules, with 10Å padding to the receptor surface. A pre-equilibrated 1-palmitoyl-2-oleoyl-sn-glycero-3-phosphocholine membrane was placed according to the OPM database [44]. Sodium ions were added to a final concentration of 0.15M. The temperature was set to 300K using the Nose-Hoover-Chain method and the systems were maintained at constant atmospheric pressure through the Martyna–Tobias–Klein method. The RESPA integrator was used to calculate intermolecular forces, using the standard cutoff of 9.0A. Unrestrained, all-atom molecular dynamics simulations were performed in triplicates using Desmond (v5.5; Schroedinger, Schroedinger, LLC, New York City, NY, USA) and the OPLS-AA force field. Simulations were performed on the cluster of the molecular design lab, FU Berlin on graphics processing units (GPU). Simulations were run for 200 ns and 1000 distinct ligand–receptor conformations were saved. VMD (v1.9.3.; Champaign, IL, USA) was used for analysis of the resulting trajectories [45]. MD-based distances were processed and transformed with pandas 1.1.2 using Python 3.7 [46]. Jointplots were created with seaborn 0.11.0 and matplotlib 3.3.2 [47,48].

Dynamic 3D-pharmacophores: Dynamic pharmacophores (dynophores) is a fully-automated combination of three-dimensional chemical feature-based pharmacophore models with dynamics information gained during MD simulations as described recently [49,50,51]. Throughout the MD trajectory, interactions are grouped into so-called superfeatures according to the type of interaction (e.g., hydrogen bond donor or hydrophobic contact) and which atoms are involved on the ligand side. The resulting superfeatures are represented as point density clouds, providing a visualization of the spatial distribution and statistical occurrence of each [30]. The dynophore method is implemented in the LigandScout framework [41,42].

## 5. Conclusions

We transferred an established concept from the muscarinic receptor field to serotonin receptors and showed that β-arrestin-biased ligands like LSD and ergotamine constrain the closure of the extracellular binding site. Since extra- and intracellular receptor regions are allosterically coupled, this also affects the receptor conformation at the intracellular side potentially resulting in differential recruitment of transducer proteins. Our data support the idea of a general mechanism for signaling bias at aminergic receptors, leading to plethora of testable hypothesis. Sound mechanistic knowledge about receptor functionality is the foundation of informed decisions in drug design. Virtual screening workflows could benefit by choosing the most relevant receptor conformation in order to identify hit molecules with a specific pharmacological profile. This might pave the way towards tailor-made therapeutics with high efficacy and reduced adverse effects.

## Supplementary Materials

Supplementary Materials can be found at https://www.mdpi.com/1422-0067/21/24/9728/s1.

## Abbreviations

GPCR	G protein-coupled receptor
TM	transmembrane domain
ECL	extracellular loop
LSD	lysergic acid diethylamide
MD	molecular dynamics
5-HT	serotonin
PDB	protein data bank
MIA	7-(4-(4-(1-Methyl-1*H*-indol-4-yl)piperazin-1-yl)butoxy)-3,4-dihydroquinolin-2(1*H*)-one
