# Supplementary material for: Biased Ligands Differentially Shape the Conformation of the Extracellular Loop Region in 5-HT2B Receptors
Source: Int J Mol Sci. 2020 Dec 20;21(24):9728. doi: 10.3390/ijms21249728 (PMC7767279; doi:10.3390/ijms21249728)
Supplement: Supplementary file 1 [file ijms-21-09728-s001.pdf]

<u>**Supporting Information for:**</u>

Biased ligands differentially shape the conformation of the extracellular loop region in serotonin receptors

Katrin Denzinger[1], Trung N. Nguyen[1], Theresa Noonan[1], Gerhard Wolber[1] and Marcel Bermudez[1]

[1]Institute of Pharmacy, Freie Universität Berlin, Königin-Luise-Strasse 2-4, 14195 Berlin, Germany

*to whom correspondence should be addressed: m.bermudez@fu-berlin.de

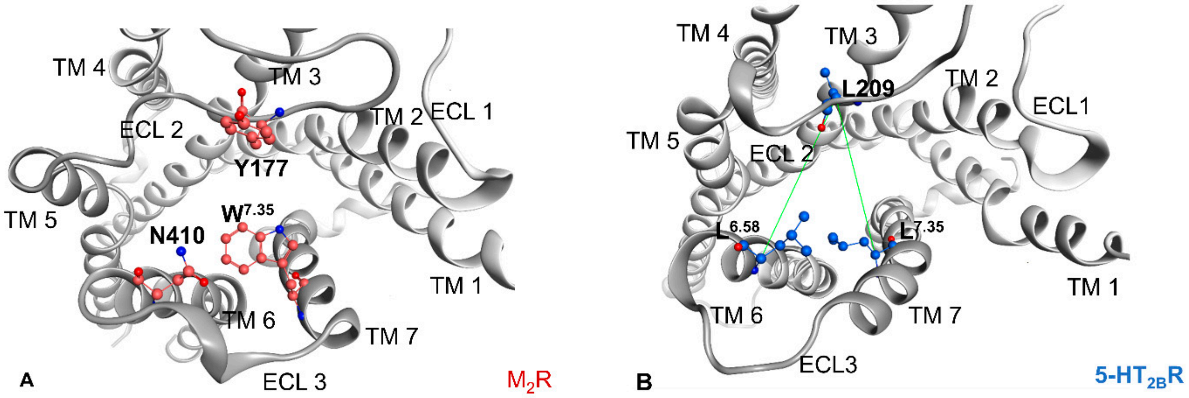

SI Figure S1: Key residues of M_2R (red) and $5HT_{2B}$ (blue) used as molecular descriptors for the examination of extracellular binding pocket closure. (**A, B**) The M_2R (**A**) served as an important model system for investigations of the correlation between the extracellular binding pocket closure and the extent of ligand bias. Cα distances between N410 in ECL3 and Y177 in ECL2 and between $W^{7.35}$ at the top of TM7 and Y122 served as molecular descriptors. The comparable residues $L^{6.58}$ at the top of TM6, $L^{7.35}$ at the top of TM7 and L209 in ECL2 (green lines) served as molecular descriptors for the extent of closure of the extracellular binding pocket at the $5HT_{2B}R$ (**B**). These distances were measured for 5-HT_{2B} bound to 5-HT, LSD, ERM, LY266097 and the apo structure for every frame of the MD simulation (see Figure S2 and S3) for this study.

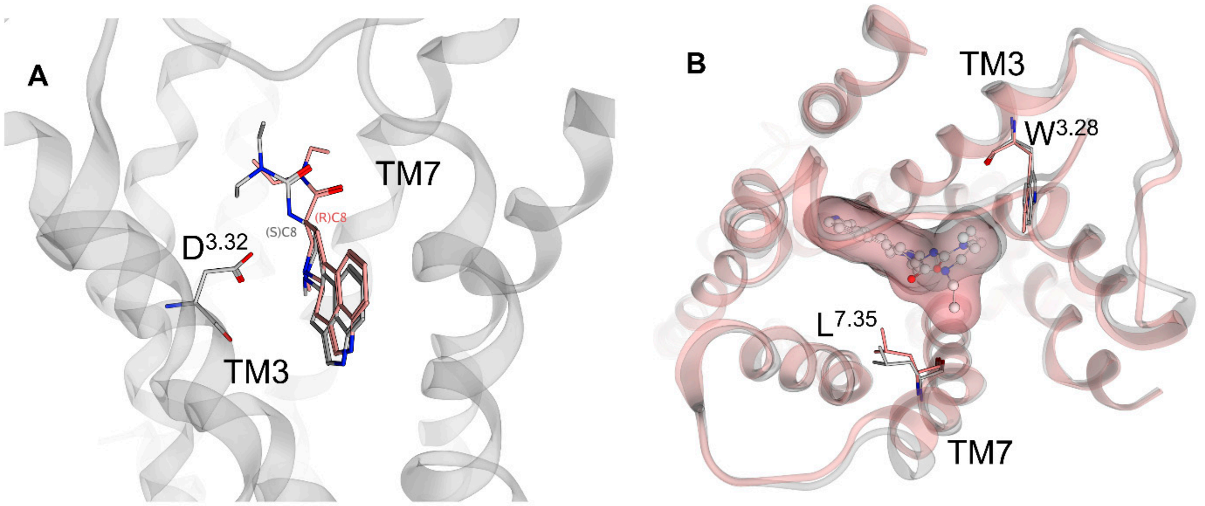

SI Figure S2: Comparison of the binding modes of LSD (**A**, salmon) and lisuride (**A**, grey) bound to the 5-HT_{2B}R (**A**, light grey ribbon). The ergoline ring system of both ligands is similarly oriented in the binding pocket. The tertiary amine points to $D^{3.32}$, a highly conserved residue for aminergic GPCR activation. The ligands differ in configuration at the C8 atom. Due to the *(S)* configuration, the diethylurea substituent of lisuride points to TM3. In contrast, the diethylamide substituent of LSD points to helix seven due to the C8 *(R)* configuration.

The extracellular view of the aligned 5-HT_{2B} receptors bound to LSD (**B**, salmon) and lisuride (**B**, grey) shows a slightly narrower extracellular loop region for the LSD-bound structure. The top of TM7 is slightly turned inward in the LSD bound structure compared to the lisuride bound structure. One ethyl moiety of LSD is oriented towards TM7 for which a lipophilic contact to $L^{7.35}$ was identified in 52% of investigated MD simulation frames (see Figure 2F). The diethylurea substituent of lisuride points to TM3 and displays close proximity to $W^{3.28}$

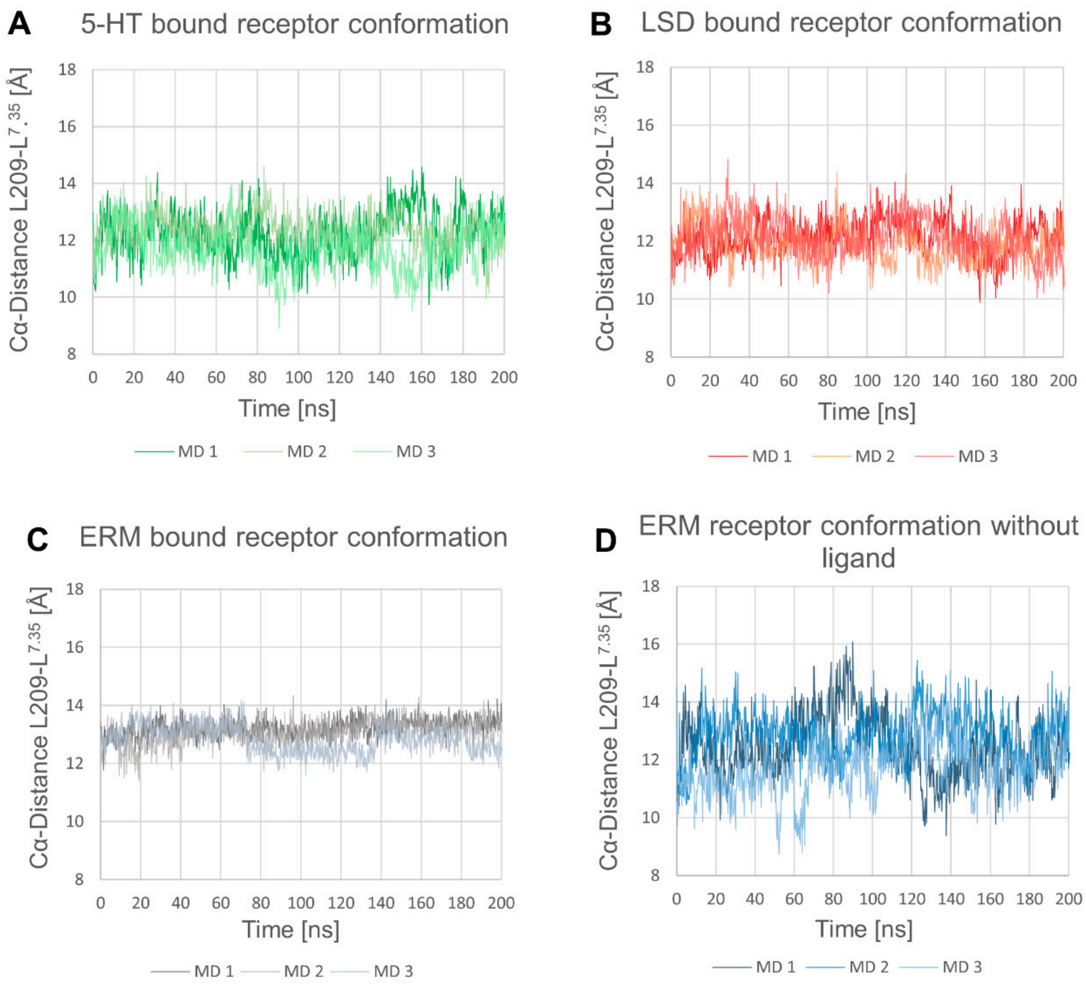

SI Figure S3: Ligand dependent Cα-distance change of L209-L$^{7.35}$ at 5-HT$_{2B}$R. The graphs show Cα distance changes during MD trajectories of the 5-HT (**A**), LSD (**B**), ERM (**C**) bound 5-HT$_{2B}$R and the apo structure of the ERM-receptor complex (**D**). Every simulation was performed in triplicates. The ERM bound receptor conformation clearly fluctuates less than the LSD and 5-HT bound conformation. The highest fluctuation can be observed for the apo structure. This suggests that the three ligands differentially constrain binding pocket closure.

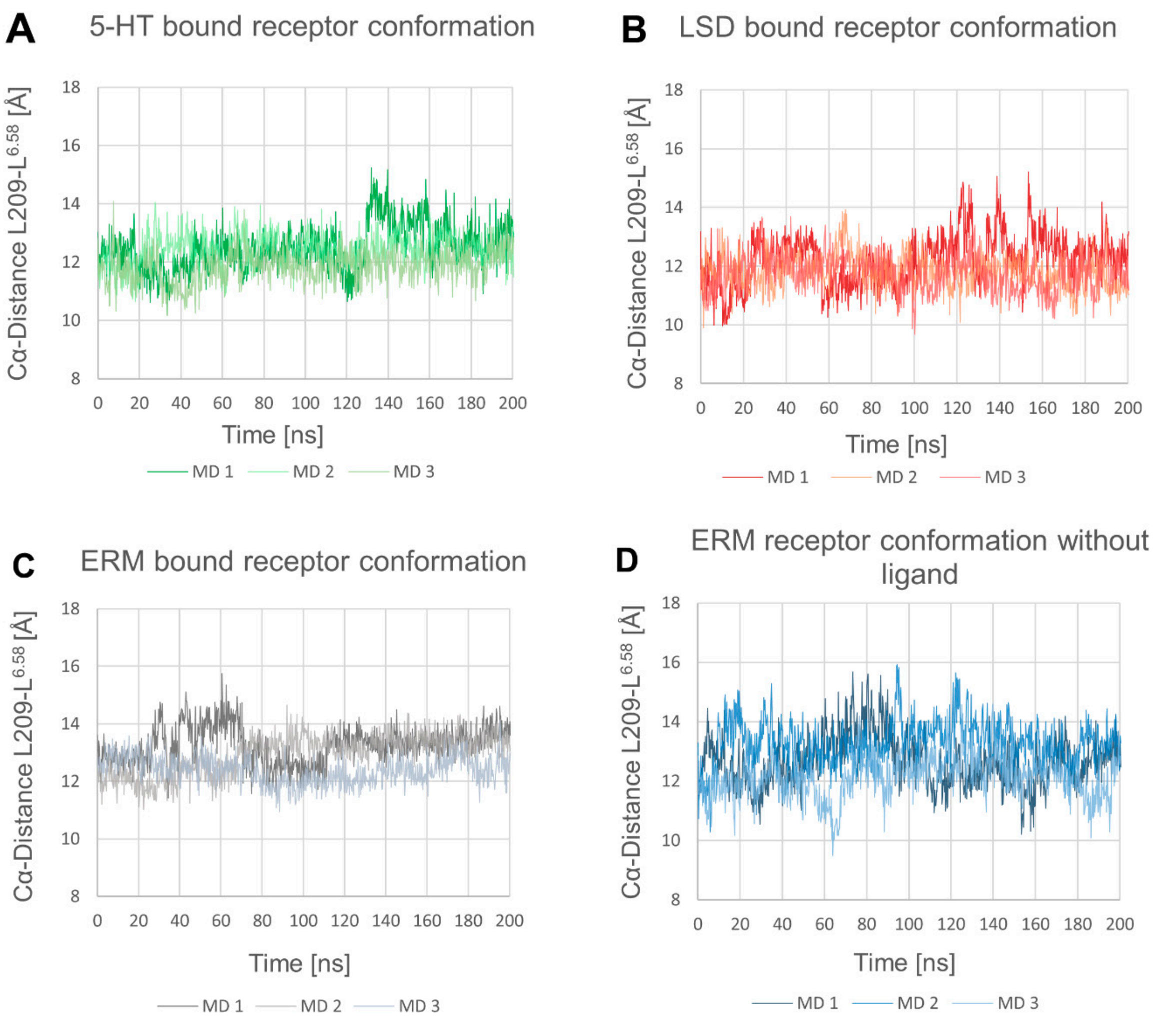

SI Figure S4: Ligand dependent Cα-distance change of L209-$L^{6.58}$ at 5-HT_{2B}R**.** The graphs show Cα-distance changes during MD trajectories of the 5-HT (**A**), LSD (**B**), ERM (**C**) bound 5-HT_{2B}R and the apo structure of the ERM-receptor complex (**D**). Every simulation was performed in triplicates. The ERM bound receptor conformation clearly fluctuates less than the LSD and 5-HT bound conformation. The highest fluctuation can be observed for the apo structure. This suggests that the three ligands differentially constrain binding pocket closure.

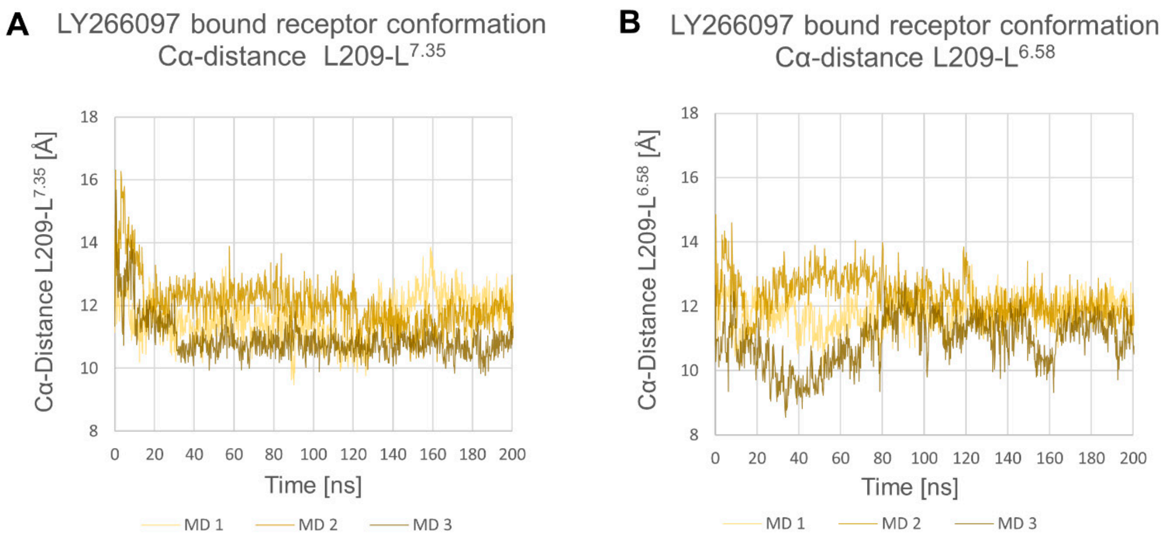

SI Figure S5: MD simulation of the LY266097- 5-HT$_{2B}$R complex. The graphs show Cα distance changes during MD trajectories of the LY266097-5-HT$_{2B}$R complex. Every simulation was performed in triplicates. Cα distance L209-L$^{7.35}$ (**A**) shows a clear decrease within the first 20 ns of the simulation. The Cα distance of L209-L$^{6.58}$ during MD simulations is shown in **B**.

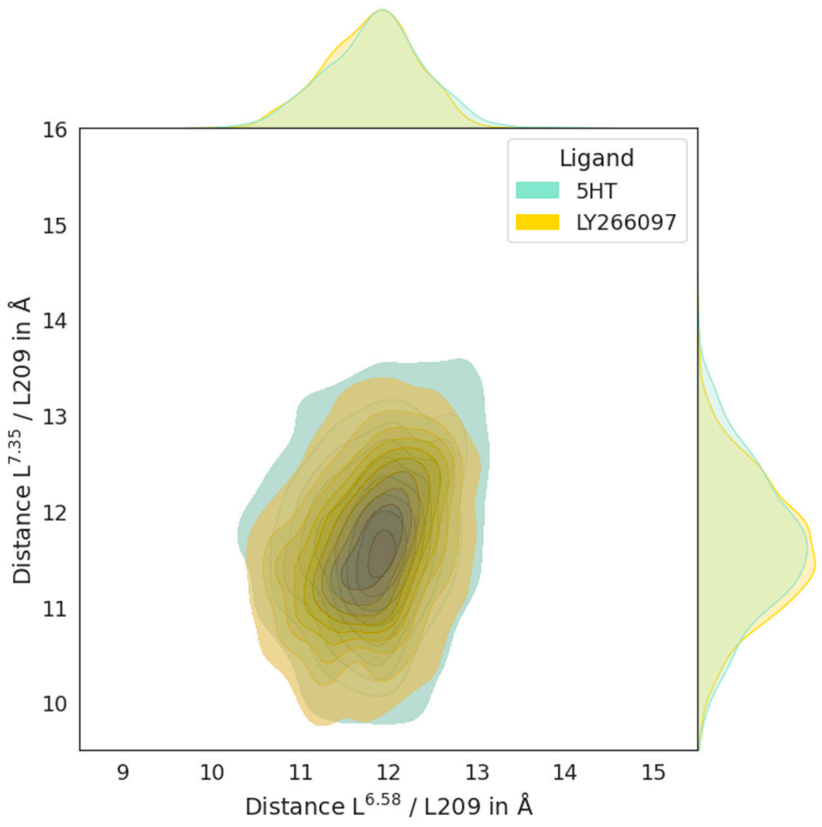

SI Figure S6: The density plot of the Cα distances between L209 and $L^{6.58}$ and $L^{7.35}$ indicates a similar distribution of the conformational descriptors in 5-HT and LY266097-bound 5-HT_{2B}R. Therefore, in contrast to the ligands in Figure S2 and S3, it is not possible to use these Cα-distances as descriptors for ligand bias.

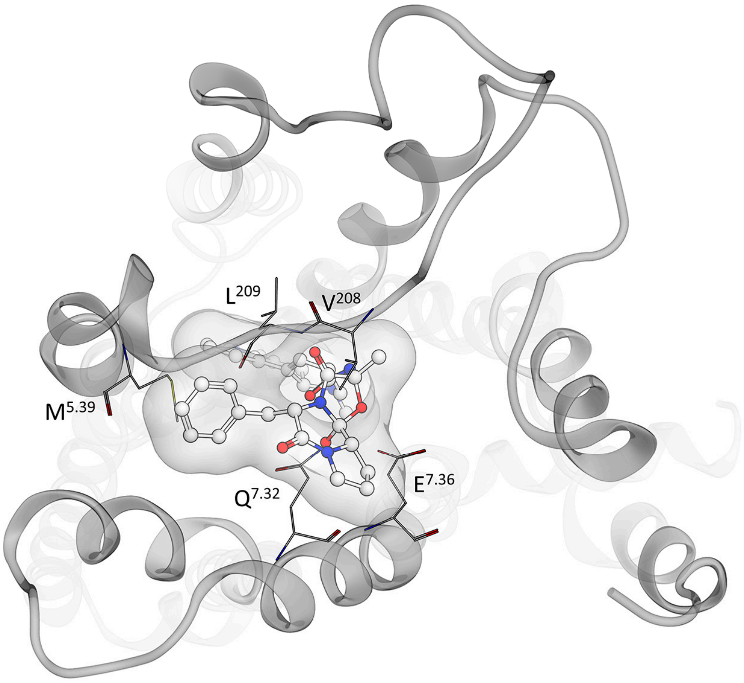

SI Figure S7: Docking of ERM into the ligand-free LSD receptor conformation resulted in clashes to residue $Q^{7.32}$, $E^{7.36}$, V208, L209 and $M^{5.39}$. The LSD bound receptor conformation exhibits a tighter binding pocket, due to less conformational ligand restriction than the ergotamine-bound conformation. Thus, the binding pocket of the LSD-bound receptor conformation doesn't provide plausible docking poses for ergotamine-like structures.
